# Measurement of Melting Point and Radiance Temperature (at Melting Point and at 653 nm) of Hafnium-3 (wt. %) Zirconium by a Pulse Heating Method*

**DOI:** 10.6028/jres.080A.065

**Published:** 1976-08-01

**Authors:** A. Cezairliyan, J. L. McClure

**Affiliations:** Institute for Materials Research, National Bureau of Standards, Washington, D.C. 20234

**Keywords:** Hafnium, high-speed measurement, high temperature, melting point, pyrometry, radiance temperature

## Abstract

A subsecond duration pulse heating method is used to measure the melting point and radiance temperature (at 653 nm) at the melting point of hafnium containing 3.12 weight percent zirconium. The results yield a value of 2471 K for the melting point on the International Practical Temperature Scale of 1968. The radiance temperature (at 653 nm) of this material at its melting point is 2236 K, and the corresponding normal spectral emittance is 0.39. Estimated inaccuracies are: 10 K in the melting point and in the radiance temperature, and 5 percent in the normal spectral emittance.

## 1. Introduction

A subsecond-duration pulse heating technique was used earlier to measure the melting point and the radiance temperature^1^ at the melting point of several refractory metals [1–7].^2^ In the present study, the same technique is used for similar measurements on hafnium containing 3.12 weight percent zirconium.

The method is based on rapid resistive self-heating of the specimen from room temperature to high temperatures (above 1500 K) in less than one second by the passage of an electrical current pulse through it; and on measuring, with millisecond resolution, such experimental quantities as current through the specimen, potential drop across the specimen, and the specimen temperature. Temperature is measured with a high-speed photoelectric pyrometer [8], which permits 1200 evaluations of specimen temperature per second. Details regarding the construction and operation of the measurement system, the methods of measuring experimental quantities, and other pertinent information, such as the formulation of relations for properties, error analysis, etc. are given in earlier publications [9, 10].

## 2. Measurements

The material used in this study was a hafnium alloy containing 3.12 (wt. %) zirconium^3^ (referred to in this paper as hafnium-3 (wt. %) zirconium. The alloy specimens were 99.97 percent pure and contained, according to the manufacturer’s analysis, the following impurities in ppm by weight: C, 15; O, 10; N, 10; Al, 20; Ca, 10; Cu, 10; Fe, <50; Mn, 20; Mo, 10; Nb, 30; Si, 20; Ta, 30; Ti, 10; W, 10. The total amount of all other detected elements was less than 70 ppm, each element being below 10 ppm limit.

Measurements were performed on two specimens in the form of tubes and nine specimens in the form of strips. The tubes, used to determine the melting point, were fabricated from rods by removing the center portion using an electro-erosion technique. A small rectangular hole (1 × 0.5 mm) was fabricated in the wall at the middle of the specimen to approximate blackbody conditions for the pyrometric temperature measurements. Prior to the experiments, the tubular specimens were heat treated by subjecting them to 10 heating pulses [up to 1700 K] in a vacuum environment of 1.3 × 10^3^ N·m^−2^ (~10^−5^ torr). The nominal dimensions of the tubes were: length, 89 mm; outside diameter, 6.4 mm; wall thickness, 0.5 mm.

The strips were used to measure the radiance temperature at the melting point. Before the experiments, the surface of the specimens was treated using abrasive; three different grades of abrasive were used yielding specimens with three different surface roughnesses (ranging from approximately 0.2 *μ*m to 0.5 *μ*m in RMS). The nominal dimensions of the strips were: length, 76 mm; width, 3.2 mm; thickness, 1.6 mm.

The experiments on the tubular specimens were conducted in a vacuum environment of approximately 1.3 × 10^3^ N·m^−2^ (~10^−5^ torr), while all the experiments on strip specimens were conduced in an argon environment at atmospheric pressure. The heating rates for the tubular specimens were approximately 8000 K·s^−1^ (specimen I) and 6000 K·s^−1^ (specimen II) corresponding to specimen heating periods (from room temperature to melting point) of approximately 300 ms and 400 ms, respectively. The heating rates for the strips near the melting point varied from 2000 K·s^−1^ to 4800 K·s^−1^, and the corresponding heating periods varied from 800 ms to 350 ms.

The high-speed pyrometer was calibrated before the entire set of experiments, using a tungsten filament standard lamp, which in turn was calibrated against the NBS “Temperature Standard.”

All temperatures reported in this paper, except where explicitly noted, are based on the International Practical Temperature Scale of 1968 [11].

## 3. Experimental Results

### 3.1 Melting Point

**Figure 1:**
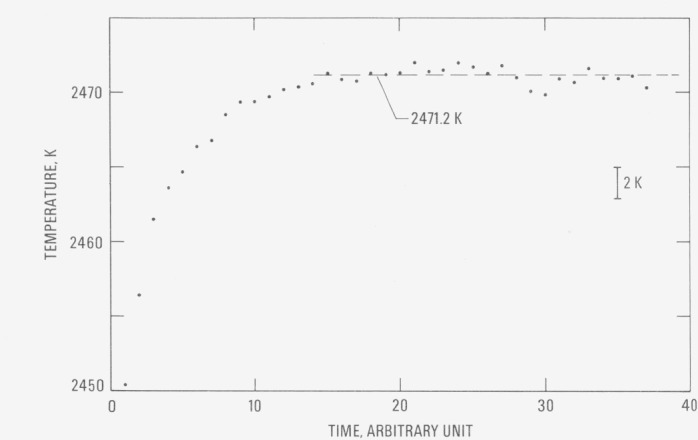
Variation of temperature of hafnium-3(wt. %) zirconium (tubular specimen I) as a function of time near and at the melting point (1 time unit = 0.833 ms).

Temperature of the tubular specimens was measured near and during the initial melting period, until the specimen collapsed. Typical results for the variation of the specimen temperature as a function of time (for specimen I) are shown in figure 1. The plateau in temperature indicates the region of solid and liquid equilibria. The complete results for the tubular specimens are presented in table 1. The value of the melting point was obtained by averaging temperature points on the plateau for each specimen. The duration of the plateau of specimen II is shorter than that of specimen I because of an early collapse of the second specimen. To determine the trend of measured temperatures at the plateau, temperatures for specimen I were fitted to a linear function in time using the least squares method. The slope of the linear function was −23 K·s^−1^, which corresponds to a maximum temperature difference of less than 0.4 K between the beginning and the end of the plateau. This procedure gave a standard deviation of 0.4 K, the same as that obtained by averaging the temperatures. The average melting point of the specimens is 2471.2 K with an average absolute deviation from the mean of 0.1 K. It may be concluded that the melting point of hafnium-3 (wt. %) zirconium measured in this work is 2471 K.

### 3.2 Radiance Temperature at the Melting Point

Radiance temperature measurements were performed on strips at 653 nm which corresponds to the effective wavelength of the pyrometer’s interference filter. The bandwidth of the filter was 10 nm. The circular area viewed by the pyrometer was 0.2 mm in diameter.

**Figure 2:**
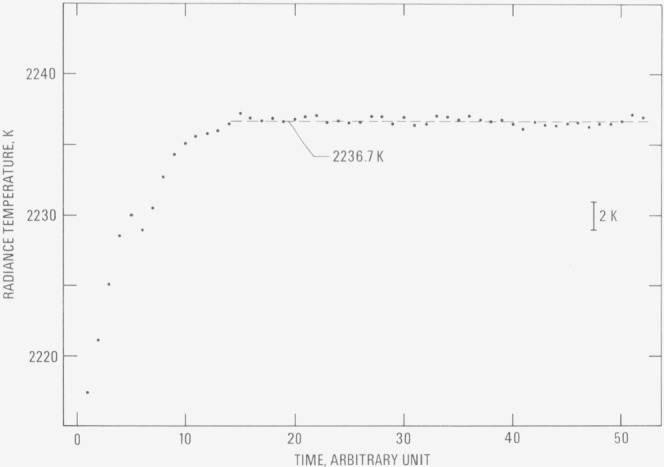
Variation of radiance temperature (at 653 nm) of hafnium-3 (wt. %) zirconium (strip specimen 2) as a function of time near and at its melting point (1 time unit =0.833 ms).

Radiance temperature of hafnium-3 (wt. %) zirconium at its melting point for the nine experiments (corresponding to nine specimens) and other pertinent results are reported in table 2. The variation of radiance temperature as a function of time near and at the melting point is shown in figure 2 for a typical experiment (specimen II).

A single value for the radiance temperature at the plateau for each specimen was obtained by averaging the temperatures at the plateau. The number of temperatures used for averaging ranged from 22 to 55, depending both on the heating rate and on the behavior of the specimen during melting. The standard deviation of an individual temperature from the average was in the range 0.1 to 0.5 K for all the experiments. Similar values (for standard deviation) were obtained when data in the premelting period were fitted to a quadratic function in time. This indicates that during melting no undesirable effects took place, such as vibration of the specimen, development of hot spots in the specimen and random changes in the specimen surface conditions.

To determine the trend of measured temperatures at the plateau, temperatures for each experiment were fitted to a linear function in time using the least squares method. The detailed results are reported in table 2. The temperature difference between the beginning and the end of the plateau (corresponding to the slope in the plateau) is in the range 0–0.7 K. The standard deviation of an individual temperature from the linear function was approximately the same as the standard deviation obtained by direct averaging of the temperatures.

**Figure 3:**
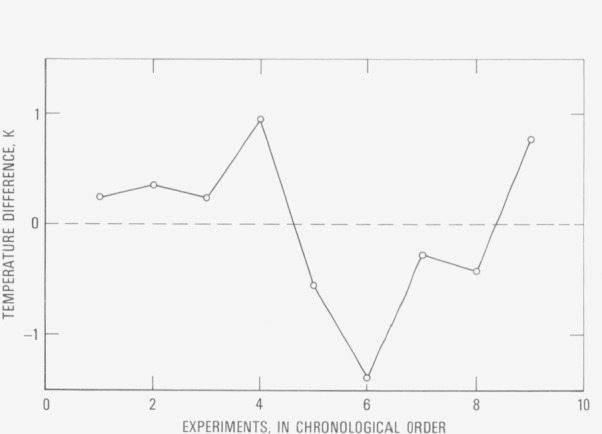
Difference of radiance temperature (at the melting point at 653 nm) of hafnium-3 (wt. %) zirconium for individual experiments from their average value of 2236.4 K (represented by the dashed line).

The average radiance temperature at the melting point for the nine specimens was 2236.4 K with an average absolute deviation of 0.6 K and a maximum absolute deviation of 1.3 K. The results are presented in figure 3. It may be concluded that the radiance temperature (at 653 nm) of hafnium- 3 (wt. %) zirconium at its melting point is 2236 K.

The normal spectral emittance at the melting point was determined using the results of the radiance temperature (obtained from the measurements on strip specimens) and the melting point (obtained from the measurements on tubular specimens). The results yield a value of 0.39 for the normal spectral emittance (at 653 nm) at the melting point of hafnium-3 (wt. %) zirconium. No effect was noticed with the variation in initial roughness.

### 3.3 Estimate of Errors

Sources and estimates of errors in experiments similar to the ones conducted in this study are given in detail in earlier publications [1, 9]. Specific items in the error analysis were recomputed whenever the present conditions differed from those in the earlier publications. The resultant estimated maximum errors in the reported values are: 10 K in the melting point and in the radiance temperature at the melting point, and 5 percent in the normal spectral emittance.

## 4. Discussion

The values of the melting point of hafnium reported in the literature are given in table 3. The values cannot be easily compared to one another or to the present value because of the differences in zirconium content of the hafnium used by the various investigators. Ackermann and Rauh [19] have estimated that the melting point of 100 percent pure hafnium should be approximately 2495 K. If the present value were corrected for the zirconium content of the hafnium, it would be somewhat higher than the estimate of Ackermann and Rauh.

No values for the normal spectral emittance of hafnium at its melting point were found in the literature. Peletskii and Druzhinin [20] have reported a value of 0.39 (at 0.65 *μ*m) at 2150 K for the *β*-phase hafnium, which is identical to the value of the present work.

## Figures and Tables

**Table 1:** Summary of measurements of the melting point of hafnium-3 (wt. %) zirconium

Specimen number	Number of temperature readings at plateau	Melting point (K)	Standard deviation (K)
			
I	22	2471.2	0.4
II	10	2471.1	.3

**Table 2:** Summary of measurements of the radiance temperature (at 653 nm) of hafnium-3 (wt. %) zirconium during melting

Specimen number^a^	Surface roughness^b^	Premelting period		Melting period				
		Heating rate^c^ (K·s^−1^)	Standard deviation^d^ (K)	Number of temperature reading^e^	Slope at plateau^f^ (K·s^−1^)	Plateau temp, difference^g^ (K)	Radiance temperature^h^ (K)	Standard deviation^i^ (K)
								
1	A	4100	0.2	22	38.1	0.7	2236.6	0.3
2	A	4800	.3	34	−13.7	−.4	2236.7	.2
3	C	3800	.2	24	0	0	2236.6	.1
4	B	3600	.2	44	−11. 5	−.4	2237.3	.4
5	A	2000	.4	49	−27. 1	−1.1	2235.8	.5
6	B	2500	.2	34	−22. 7	−0.6	2235.0	.3
7	C	2500	.4	42	−5. 9	−.2	2236.1	.2
8	A	2200	.2	55	−7. 8	−.4	2236.0	.3
9	B	3600	.2	36	−6. 8	−.2	2237.1	.3

a Also represents the experiments in chronological order.

b The notations used for surface conditions correspond to the following typical roughnesses in *μ*m A, 0.2; B, 0.4; C, 0.5.

c Heating rate evaluated at a temperature approximately 10 K below the melting point.

d Represents standard deviation of an individual temperature as computed from the difference between the measured value and that from the smooth temperature versus time function (quadratic) obtained by the least squares method. Data extend approximately 90 K below the melting point.

e Number of temperature readings used in averaging the results at the plateau to obtain an average value for the radiance temperature at the melting point of the specimen.

f Derivative of the temperature versus time function obtained by fitting the temperature data at the plateau to a linear function in time using the least squares method.

g Maximum radiance temperature difference between the beginning and the end of the plateau based on the linear temperature versus time function.

h The average (for a specimen) of measured radiance temperatures at the plateau.

i Standard deviation of an individual temperature as computed from the difference between the measured value and that from the average plateau radiance temperature.

**Table 3:** Melting point of hafnium reported in the literature

Investigator	Ref.	Year	Zirconium content (wt. %)	Temperature^a^ (K)
				
Litton	12	1951	0.70–0.89	2407 ±15
Deardorff and Hayes.	13	1956	0.008	2499±30^b^
Carlson, et. al	14	1957	.01	2427
Giessen, et. al	15	1962	2.3	2509 ± 35
Peterson and Beernsten.	16	1963	0.03	2467
Taylor, et. al	17	1963	2.3	2467
Krikorian and Wallace.	22	1964	<0.02	2507 ±20
Copeland and Goodrich.	18	1969	.2	2463
Ackermann and Rauh.	19	1971	.7	2467
Present work			3.12	2471 ±10

a All temperatures reported in the original references published prior to 1968 are converted to IPTS-68.

b In 1965 Deardorff, et. al. [21] reported a revision of this value which when corrected to IPTS-68 is 2467 ± 15K.

## Footnotes

1 Radiance temperature (sometimes referred to as brightness temperature) is the apparent temperature of the specimen surface corresponding to the effective wavelength of the measuring pyrometer.

2 Figures in brackets indicate the literature references at the end of this paper.

3 The analysis to determine the zirconium content was performed by the Spectrochemical Analysis Section at NBS.
